# Evaluation of Central Venous Catheter for Dialysis Associated with Bloodstream Infections

**DOI:** 10.3400/avd.oa.23-00062

**Published:** 2023-12-15

**Authors:** Erwin Hadi Chandra, Tom Ch. Adriani, Ahmadi Alwi, Mulawardi, Nyityasmono Tri Nugroho, Dewi Yusuf

**Affiliations:** 1 Division for Vascular and Endovascular, Department of Surgery, Faculty of Medicine, Hasanuddin University, Dr. Tadjuddin Chalid National Hospital, Makassar, Indonesia; 2 Department of Surgery, Wahidin Sudirohusodo Hospital, Faculty of Medicine, Hasanuddin University, Makassar, Indonesia; 3 Division for Vascular and Endovascular, Department of Surgery, Faculty of Medicine, Universitas Indonesia, Cipto Mangunkusumo National Hospital, Jakarta, Indonesia; 4 Division for Vascular and Endovascular, Stella Maris Hospital, Makassar, Indonesia

**Keywords:** catheter-related bloodstream infections (CRBSIs), hemodialysis, central venous catheter, end-stage renal disease

## Abstract

**Objective**: Hemodialysis (HD) catheter-related bloodstream infections (CRBSIs) are a major complication of long-/short-term catheter.

**Material and Methods**: Patients with HD CRBSIs were identified, and their blood was taken and sent to clinical pathology for culture and sensitivity testing. The inclusion criteria were adults with end-stage renal disease who required urgent HD access in the presence of a central venous catheter (CVC) infection.

**Results**: The most common isolates among the patients with CRBSIs were gram-positive microorganisms (57.5%) and gram-negative organisms (42.5%). Overall, in our entire study, *Staphylococcus aureus* was the most common pathogen isolated, accounting for 30%, followed by *Pseudomonas aeruginosa* (20%), coagulase-negative staphylococci (CoNS) (12.5%), *Klebsiella* spp. and *Acinetobacter* (10%), *Staphylococcus epidermidis* (7.5%), and methicillin-resistant *Staphylococcus aureus* (MRSA), *Escherichia coli*, *Staphylococcus hominis*, and *Enterococcus faecalis* (2.5%). The commonest bacterial in femoral was *S. aureus*, and for subclavian was *Pseudomonas aeruginosa*. All *S. aureus* were sensitive to aminoglycosides and quinolones. *P. aeruginosa* was sensitive to the third generation of cephalosporins, especially cefoperazone and carbapenem.

**Conclusion:** Nontunneled CVCs used for more than 2 weeks could increase the risk of CRBSIs. Procalcitonin and erythrocyte sedimentation rate could predict the CRBSIs in this study. This study also revealed that the gram-positive bacteria were *primadonna* in dialysis of CRBSIs, and most of them were sensitive to aminoglycosides.

## Introduction

Increased morbidity, mortality, and healthcare expenses are linked to catheter-related bloodstream infections (CRBSIs). When these devices are required, preventing CRBSIs is a crucial goal. The kind of catheter, the purpose for which it is used, how it is used, as well as the unique traits of the patient in whom it is inserted are all elements that determine the risk of infection.^1^^,^^2)^ The fact that CRBSIs rates vary depending on the institution and healthcare unit under examination is not surprising. In order to enhance CRBSIs’ care and prevention, this study will analyze patient characteristics, bacterial pathogen patterns, and their sensitivity to medications.

A serious clinical issue, CRBSIs, is a nosocomial infection that is always changing due to changes in the population at risk, the range of bacteria that are present, and an increase in the use of broad-spectrum antibiotics. In addition to CRBSIs, central venous catheters (CVCs) increase the risk of a patient experiencing a number of local and systemic side effects, including infection at the site of insertion, septic thrombophlebitis, endocarditis, metastatic infections, and other life-threatening conditions such as bacteremia, sepsis, and death.^3^^,^^4)^ Therefore, it is crucial that these infections are identified as soon as possible and diagnosed promptly using a combination of clinical signs and quantitative culture techniques, as well as their antibiotic susceptibility, which serves as a crucial tool in assisting the doctor in initiating the proper therapy.^4)^

The incidence of CRBSIs was 2.79 infections per 1000 catheter days in 2005, according to Lorente et al., while CVC was involved in 2.04% of cases.^5)^ Singh et al. determined that the overall infection rate for CRBSIs was 0.48 per 1000 device days.^6)^ Parameswaran et al. found that 8.75 CRBSIs occurred for every 1000 catheter days.^7)^ The reported incidence of CRBSIs varies from nation to nation and even from hospital to hospital. Bloodstream infections, according to a meta-analytical study conducted at Johns Hopkins University, are the third most common reason for hospital-acquired infections. The death rate related to these infections ranges from 12% to 25%.^8)^

## Material and Methods

Patients with hemodialysis (HD) CRBSIs were identified, and their blood was taken and sent to clinical pathology for culture and sensitivity testing. The diagnosis of CRBSIs is based on the presence of CVC, signs of catheter infection, clinical symptoms and signs of bacteremia, and a positive blood culture. The inclusion criteria were adults with end-stage renal disease (ESRD) who required urgent HD access in the presence of a CVC infection. Patients were divided into subclavian and femoral groups. All patients were asked to provide written informed consent during the initial appointment if they were willing to allow the use of their clinical data for publications or presentations in the scientific community. The protocols were carried out in compliance with the Helsinki Declaration of 1975 and the institutional and national responsible committees’ ethical standards for human experimentation. The Hasanuddin University clinical research council gave the project their blessing before it could begin (UH 22040100, May 19, 2022).

Suspected cases of CRBSIs were those patients on an indwelling central catheter who had symptoms of local phlebitis, inflammation, purulence, or both at the insertion site, along with fever, chills, hypotension, and an increased leucocyte count.^1)^ The findings were noted, which contained demographic details such as the ward the patient was admitted to, name, age, sex, date of admission, diagnosis, associated medical conditions, and so on. Additionally, information on the type of catheter used, its length, any prior catheterizations, and any infections that may have developed as a result of the temporary catheter were gathered. Additionally, total leucocyte count, fever, and any other pertinent laboratory test results were documented.

Two blood samples were taken from people who had CRBSI-related suspicions. The catheter was used for one and the peripheral site for the other. In our hospital, two-lumen polyurethane latex-free CVCs were inserted. We collected the sample. After carefully washing hands, a fresh pair of sterile gloves were put on. The catheter was then flushed with a 10-mL syringe of regular saline that had been prefilled. The first 3 to 5 mL of blood were taken out and thrown away. The 50-mL BACTEC bottles were then filled with the blood sample (5 mL), which would later be processed on BD BACTEC FX40 equipment.

**Figure 1:**
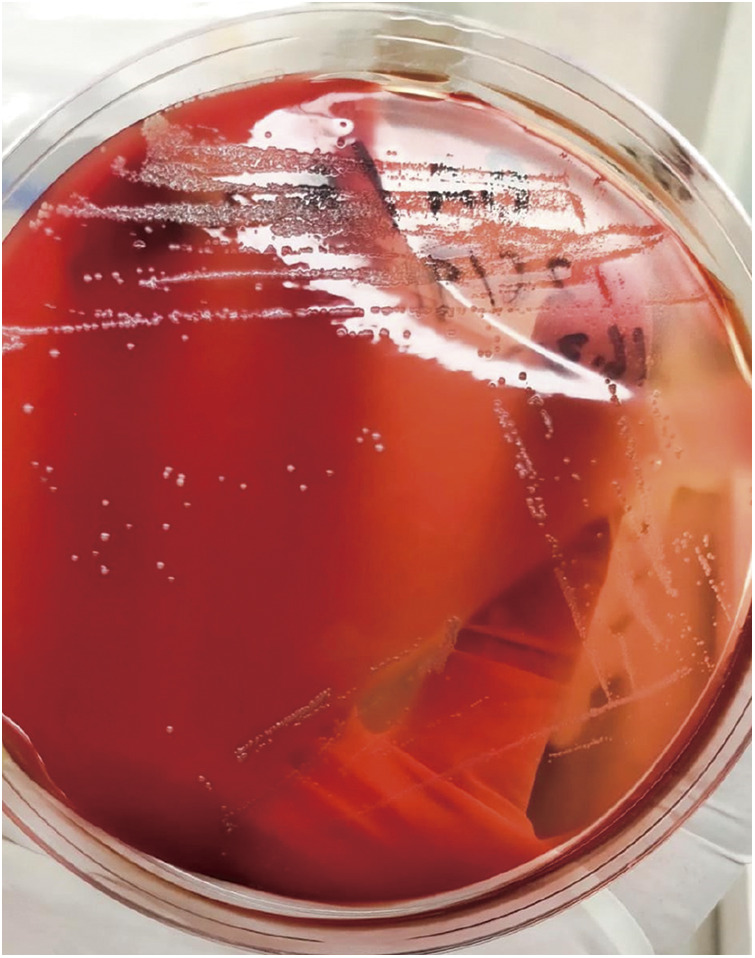
Fig. 1 Plate exhibiting colonies on blood agar for diagnosis of CRBSIs. CRBSIs: catheter-related bloodstream infections

Upon discovering bacterial growth on the bottle, a sample was taken for Gram staining and processing using a 1-mL needle that was inserted into the rubber cap of the bottle. On several culture medium plates, an aliquot was cultivated in part. For 24 hours, the plates were incubated at 37°C. Following incubation, the plates were checked for any development; the colonies (**Fig. 1**), if any, were stained with a Gram stain; and the organisms were identified using a variety of biochemical assays.

## Results

Twenty patients were inserted with subclavian HD CVC and 20 patients were inserted with femoral CVC. The demographic study showed (**Table 1**) 55% were male (22/40), and the female study showed 45% (18/40) and an age of 45.5 ± 11.1 years. The duration of catheter insertion in the subclavian and femoral groups was 52.9 ± 42.6 days and 38 ± 10.5 days, respectively. The length of hospital stay in the subclavian and femoral groups was 10 ± 6.4 days and 9.4 ± 7.1 days, respectively. Based on SIRS indications, the pulse rate, respiration, temperature, and white blood cell (WBC) count for the subclavian group were 88.2 ± 7.6 x/min, 19.7 ± 3.7 x/min, 37 ± 0.6°C, and 11.4 ± 5.2 × 10^3^/µL with p values of 0.60, 0.146, 0.361, and 0.025, respectively, and for the femoral group, these values were 86.7 ± 10.3 x/min, 21.1 ± 2.3 x/min, 38.1 ± 0.4°C, and 14.7 ± 4.0 × 10^3^/µL, respectively. Procalcitonin, lactate, and erythrocyte sedimentation rate (ESR) values in the subclavian group were 67.7 ± 64.2 µg/L, 3.7 ± 1.4 mmol/L, 84 ± 24.1 mm/h, respectively, and these values in the femoral group were 111 ± 47 µg/L, 3.6 ± 0.8 mmol/L, and 105 ± 24.1 mm/h, respectively, with p values in both groups being 0.02, 0.68, and 0.004, respectively. Creatinine and urea values in the subclavian group were 7.8 ± 4.8 mg/dL and 133 ± 99 mg/dL, respectively, and these values in the femoral group were 6.6 ± 3.2 mg/dL and 91.5 ± 34.1 mg/dL, respectively. The length of treatment in the subclavian and femoral groups was 9.9 ± 6.3 and 9.3±7.1 days, respectively.

**Table:** Table 1 Subgroup analyses of time to catheter colonization

Variable	Femoral	Subclavia	p value
Sex	–	–	–
Male	7	15	–
Female	13	5	–
Catheter duration (mean)	38 ± 10.5 days	52.9 ± 42.6 days	–
Length of hospital stay	9.4 ± 7.1	10 ± 6.4	–
White blood cell differentiation (mean)	14.7 ± 4.0 × 10^3^ µL	11.4 ± 5.2 × 10^3^ µL	0.025
Body temperature	38.1 ± 0.4°C	37 ± 0.6°C	0.361
Heart rate	86.7 ± 10.3 x/min	88.2 ± 7.6 x/min	0.6
Respiration	21.1 ± 2.3 x/min	19.7 ± 3.7 x/min	0.146
Procalcitonin	111 ± 47 µg/L	67.7 ± 64.2 µg/L	0.020
Lactate	3.6 ± 0.8 mmol/L	3.7 ± 1.4 mmol/L	0.680
ESR	105 ± 24.1 mm/h	84 ± 24.1 mm/h	0.004
Creatinine	6.6 ± 3.2 mg/dL	7.8 ± 4.8 mg/dL	–
Ureum	91.5 ± 34.1 mg/dL	133 ± 99 mg/dL	–

ESR: erythrocyte sedimentation rate

The most common microorganisms found in CRBSIs were gram positive (**Table 2**) (57.5%) and gram negative (42.5%). Overall, *Staphylococcus aureus* was the most common pathogen found, with a value of 30%, followed by *Pseudomonas aeruginosa* (20%), coagulase-negative staphylococci (CoNS) (12.5%), *Klebsiella* spp. and *Acinetobacter* (10%), *Staphylococcus epidermidis* (7.5%), and methicillin-resistant *Staphylococcus aureus* (MRSA), *Escherichia coli*, *Staphylococcus hominis*, and *Enterococcus faecalis* (2.5%). The most common bacteria found at the femoral level was *S. aureus*, and at the subclavian insertion, it was *P. aeruginosa*. As for antibiotic sensitivity information as shown (**Table 3**), *S. aureus* was sensitive to aminoglycosides and quinolones. *P. aeruginosa* was sensitive to the third class of cephalosporins, especially cefoperazone and carbapenem. CoNS *Klebsiella* spp. was sensitive to quinolones; *Klebsiella* spp. was sensitive to aminoglycosides, especially gentamicin; and *Acinetobacter* was sensitive to third-class cephalosporins, quinolones, and aminoglycosides. *S. epidermidis* was sensitive to quinolones and vancomycin, MRSA was sensitive to vancomycin, *E. coli* was sensitive to third-generation cephalosporins and carbapenems, *S. hominis* was sensitive to aminoglycosides and vancomycin, and *E. faecalis* was sensitive to vancomycin. We also found that there was no significant association between bacterial infections in the femoral and subclavian groups with a p value of 0.164 and no significant association between antibiotics in CRBSIs in the subclavian and femoral groups with a p value of 0.210.

**Table:** Table 2 Isolated microorganism found on CRBSIs

Miroorganism	Number of cases
*Staphylococcus aureus*	12 cases (30%)
*Pseudomonas aeruginosa*	8 cases (20%)
CoNS	5 cases (12.5%)
*Klebsiella*	4 cases (10%)
*Acinetobacter*	4 cases (10%)
*Staphylococcus epidermidis*	3 cases (7.5%)
MRSA	1 cases (2.5%)
*Escherichia coli*	1 cases (2.5%)
*Staphylococcus hominis*	1 case (2.5%)
*Enterococcus faecalis*	1 case (2.5%)

CRBSIs: catheter-related bloodstream infections; CoNS: coagulase-negative staphylococci; MRSA: methicillin-resistant *Staphylococcus aureus*

**Table:** Table 3 Antibiotic susceptibility microorganism on CRBSIs

Antibiotic	*Staphylococcus aureus* (12)	*Pseudomonas aeruginosa* (8)	CoNS (5)	*Klebsiella* (4)
Cefazolin	9 (75%)	7 (87.5%)	3 (60%)	1 (25%)
Cefoperazone	8 (66.7%)	8 (100%)	4 (80%)	1 (25%)
Meropenem	11 (91.7%)	6 (75%)	4 (80%)	3 (75%)
Gentamicin	11 (91.7%)	7 (87.5%)	4 (80%)	3 (75%)
Oxacillin	8 (66.7%	3 (37.5%)	3 (60%)	3 (75%)
Ciprofloxacin	11 (91.7%)	6 (75%)	4 (80%)	2 (50%)
Levofloxacin	11 (91.7%)	6 (75%)	5 (100%)	1 (25%)
Moxifloxacin	11 (91.7%)	5 (62.5%)	5 (100%)	2 (50%)
Erythromycin	8 (66.7%)	3 (37.5%)	3 (60%)	1 (25%)
Vancomycin	9 (75%)	6 (75%)	4 (80%)	2 (50%)

CRBSIs: catheter-related bloodstream infections; CoNS: coagulase-negative staphylococci

## Discussion

In this study, it was found that CRBSIs were mostly found at the age of 4 decades. Repeated CVC insertion and a long duration of CVC use are risk factors for CRBSIs. Based on The National Kidney Foundation’s Kidney Disease Outcomes Quality Initiative (KDOQI) 2019, it states that nontunneled central venous catheters (NT-CVCs) can be used in emergencies with a maximum duration of 2 weeks due to the increased risk of infection, and are only used in emergencies.^1)^ In this study, it was found that the use of catheters with a duration that exceeds the duration that should be HD CVC insertion can be done in three places, namely, the internal jugular, femoral, and subclavian. To reduce the risk of infection, access through the internal jugular should be used, but in this study there was no insertion in the internal jugular. In this study, several insertions use ultrasound guidance, whereas based on the research of Aydin et al., the use of ultrasound guidance can reduce the risk of complications such as insertion in the artery.^8)^ During the study period, 40 patients presented at our institution, male 22 patients (55%) and female 18 patients (45%), with the femoral group 20 patients and the subclavian group 20 patients. The catheter duration for the femoral group was 38 ± 10.5 days and that for the subclavia group was 52.9 ± 42.6 days.

Procalcitonin, lactate, and ESR in the subclavian group were 67.7 ± 64.2 μg/mL, 3.7 ± 1.4 mmol/L, and 84 ± 24.1 mm/h, respectively, and in the femoral group, they were 111 ± 47 μg/mL, 3.6 ± 0.8 mmol/L, and 105 ± 24.1 mm/h, respectively. The p values for them were 0.02, 0.68, and 0.004, respectively.

*S. aureus* was the most frequently isolated pathogen in this investigation, accounting for nearly 30% of infections linked to CRBSIs. The primary etiological agents of CRBSIs are staphylococci. *S. aureus* bacteremia is a serious illness with a high mortality rate and risk of complications.^1^^,^^2)^ *P. aeruginosa* and CoNS were the next most common isolates discovered. *P. aeruginosa* made up 20% and CoNS made up 12.5% in this study. According to the National Nosocomial Infections Surveillance (NNIS) system, CoNS has been the most frequently isolated pathogens, accounting for around 28% of infections caused by CRBSIs,^3)^ followed by *Klebsiella* spp. 4 cases (10%), *Acinetobacter* 4 cases (10%), *S. epidermidis* 3 cases (7.5%), MRSA 1 case (2.5%), *E. coli* 1 case (2.5%), and *S. hominis* and *E. faecalis*, each of them 1 case (2.5%). An interesting finding in this study was that *Escheria coli* was found which was not found in many other studies. Interestingly, there was a difference in the type of most bacterial colonies between femoral and subclavian. We do not have a journal to support whether there is a difference between the location of catheter insertion and the type of infecting bacteria. In our opinion, the hygiene of the patient also determines the pathogenicity of the bacteria.

Regarding this study, aminoglycosides and quinolones were effective against all strains of *S. aureus*. Particularly, cefoperazone, the third class of cephalosporins, and carbapenem were effective against *P. aeruginosa*. *Acinetobacter* is sensitive to third-class cephalosporins, quinolones, and aminoglycosides; *Klebsiella* spp. is sensitive to aminoglycosides, particularly gentamicin. *E. coli* was sensitive to third-generation cephalosporins and carbapenems, *S. aureus* was sensitive to aminoglycosides and vancomycin, and *E. faecalis* was sensitive to vancomycin. *S. epidermidis* was sensitive to quinolones and vancomycin.

## Limitation

The disadvantages of this study are the small number of samples. We also face problems regarding the installation that is not based on KDOQI regarding the installation location in the internal jugular area. This is because the hospital where the author did the residency did not have ultrasound, which is the standard for catheter insertion.

## Conclusions

Dialysis patients with CRBSIs were predominantly female in the fourth decade. Nontunneled CVCs used for more than 2 weeks could increase the risk of CRBSIs. Procalcitonin and ESR could predict the CRBSIs in this study. This study also revealed that the gram-positive bacteria were *primadonna* in dialysis of CRBSIs, and most of them were sensitive to aminoglycosides. CRBSIs are a problem that can cause significant morbidity, mortality, excess length of stay, and cost. KDOQI Guideline considers to limit the use of temporary CVC to maximun of 2 weeks due to increase risk of infection and should be considered in patients in need of emergent access. KDOQI considers reasonable tunneled CVC is the most appropriate permanent dialysis access and there is no maximum time limit to CVC use.

## Ethics

- Appropriate written informed consent was obtained from the patient for effect and for publication of this original article.
- This study has received ethical approval review for research.

## Disclosure Statement

All authors declare no conflict of interest.

## Author Contributions

Study conception: EHC and TCA

Data collection: EHC and TCA

Analysis: all authors

Manuscript preparation: EHC and TCA

Funding acquisition: none declared

Critical review and revision: all authors

Final approval of the article: all authors

Accountability for all aspects of the work: all authors.
